# Differential protection against oxidative stress and nitric oxide overproduction in cardiovascular and pulmonary systems by propofol during endotoxemia

**DOI:** 10.1186/1423-0127-16-8

**Published:** 2009-01-15

**Authors:** Yen-Chin Liu, Alice YW Chang, Yu-Chuan Tsai, Julie YH Chan

**Affiliations:** 1 Department of Biological Science and Center for Neuroscience, National Sun Yat-sen University, Kaohsiung, Taiwan; 2 Department of Anesthesiology, College of Medicine, National Cheng Kung University, Tainan, Taiwan; 3 Department of Medical Education and Research, Kaohsiung Veterans General Hospital, Kaohsiung, Taiwan

## Abstract

**Background:**

Both overproduction of nitric oxide (NO) and oxidative injury of cardiovascular and pulmonary systems contribute to fatal cardiovascular depression during endotoxemia. We investigated in the present study the relative contribution of oxidative stress and NO to cardiovascular depression during different stages of endotoxemia, and delineated their roles in cardiovascular protective effects of a commonly used anesthetic propofol during endotoxemia.

**Methods:**

Experimental endotoxemia was induced by systemic injection of *E. coli *lipopolysaccharide (LPS, 15 mg/kg) to Sprague-Dawley rats that were maintained under propofol (15 or 30 mg/kg/h, i.v.) anesthesia. Mean systemic arterial pressure (MSAP) and heart rate (HR) were monitored for 6 h after the endotoxin. Tissue level of NO was measured by chemical reduction-linked chemiluminescence and oxidative burst activity was determined using dihydroethidium method. Expression of NO synthase (NOS) was determined by immunoblotting. The Scheffé multiple range test was used for post hoc statistical analysis.

**Results:**

Systemic injection of LPS (15 mg/kg) induced biphasic decreases in MSAP and HR. In the heart, lung and aorta, an abrupt increase in lipid peroxidation, our experimental index of oxidative tissue injury, was detected in early stage and sustained during late stage cardiovascular depression. LPS injection, on the other hand, induced a gradual increase in tissue nitrite and nitrate levels in the same organs that peaked during late stage endotoxemia. Propofol infusion (15 or 30 mg/kg/h, i.v.) significantly attenuated lipid peroxidation in the heart, lung and aorta during early and late stage endotoxemia. High dose (30 mg/kg/h, i.v.) propofol also reversed the LPS-induced inducible NO synthase (iNOS) upregulation and NO production in the aorta, alongside a significant amelioration of late stage cardiovascular depression and increase in survival time during endotoxemia.

**Conclusion:**

Together these results suggest that oxidative injury and NO may play a differential role in LPS-induced cardiovascular depression. Oxidative tissue injury is associated with both early and late stage; whereas NO is engaged primarily in late stage cardiovascular depression. Moreover, propofol anesthesia may protect against fatal cardiovascular depression during endotoxemia by attenuating the late stage NO surge in the aorta, possibly via inhibition of iNOS upregulation by the endotoxin.

## Background

Sepsis poses a major clinical problem in management of patients in the intensive care units. Sepsis carries a high mortality rate and is the leading cause of death in critically ill patients [1,2]. Most common cause of sepsis in human is a contamination of the blood with bacteria. Endotoxins of Gram-negative bacteria induce systemic inflammatory responses characterized by induction of pro-inflammatory cytokines, fever, hypotension and intravascular coagulation [3]. Uncontrolled inflammatory responses to bacteria infection result in collapse of cardiovascular functions, leading to multiple organ failure and mortality of sepsis [4,5]. Diverse molecular mechanisms of inflammation and cellular damage contribute to cardiovascular depression during sepsis, of which overt production of nitric oxide (NO) and oxidative stress of a heightened tissue level of the reactive oxygen species (ROS) have attracted intensive research because of their intimate roles in regulation of cardiovascular and pulmonary functions [6-8]. Overproduction of NO accounts in part for endotoxin-induced vascular hyporactivity and hypotension [9]. NO is also a key molecule responsible for acute pulmonary injury during endotoxemia [10,11]. ROS, in particular superoxide anion (O_2_^•-^), mediates the reduced vasoconstriction, impaired bronchodilation and endothelial dysfunction in endotoxemia [8,12]. O_2_^•- ^is also a proinflammatory mediator that is involved in recruitment of neutrophils [13,14], formation of chemotactic factors [13,15], initiation of lipid peroxidation [16], and release of proinflammatory cytokines [11,17] during endotoxemia. Although a vast amount of evidence supports NO and O_2_^•- ^in the pathological sequelae of endotoxemia, relative contribution of these two molecules to cardiovascular depression during different stages of endotoxemia has not been fully defined.

Propofol (2,6-diisopropylphenol) has gained common application in the intensive care units for sedation and hypnosis purposes bacause of its pharmacokinetics of rapid uptake and elimination from the central nervous system and short duration of action [18]. Accumulating evidence suggests that propofol possesses nonsedative protective effects against endotoxemia. Propofol exerts anti-inflammatory effects [19,20], inhibits platelet aggregation [21], suppresses neutrophil infiltartion [21,22], and improves endothelial dysfunction [23] in animal models of endotoxemia and in patients of sepsis. Propofol also inhibits oxidative damage in vascular smooth muscle cells [24]and protects vascular endothelium from oxidative injury [23]. In addition, it decreases inducible NO synthase (iNOS) activity, and inhibits NO production during endotoxemia [19,20,22,23]. All these protective effects of propofol have been reported to be beneficial to endotoxemia. Significance of the protective effects of propofol during different stages of endotoxemia, however, remains largely unexplored.

Endotoxemia induced by intravenous injection of *E. coli *lipopolysaccharide (LPS) into the laboratory animals has been used to replicate the pathophysiological events of septic shock in patients [25]. In the present study, we used different dose of propofol (15 or 30 mg/kg/h) to examine whether the anesthetic may possess differential protective effect against cardiovascular depression during different stages of LPS-induced endotoxemia by exerting differential protective effects against oxidative stress and overproduction of NO in cardiovascular and pulmonary systems.

## Methods

### Animals

Male adult (10–12 week old) Sprague-Dawley rats (250–340 g, n = 315), purchased from BioLASCO Co., Taiwan, were used. They were housed in an animal room under temperature control (24 ± 0.5°C) and 12-h light-dark (08:00 to 20:00) cycle. Standard laboratory rat chow (PMI Nutrition International, Brentwood, MO, USA) and tap water were available *ad libitum*. All animals were allowed to acclimatize for at least 7 days prior to experimental manipulations. All experimental procedures were carried out in compliance with the guidelines of our institutional animal care committee, and were in accordance with the Guide for the Care and Use of Laboratory Animals as adopted and promulgated by the U.S. National Institutes of Health.

### General preparation

Rats were anesthetized initially with pentobarbital sodium (50 mg/kg, ip) to perform preparatory surgery [26], which included intubation of the trachea to facilitate ventilation and cannulation of the femoral artery and vein for systemic arterial pressure (SAP) measurement and drug administration. All surgical procedures were performed under a surgical plane of anesthesia as indicated by the absence of withdrawal reflex to hindpaw pinch. Pulsatile or mean SAP (MSAP) and heart rate (HR) were recorded on a polygraph (Gould, Valley View, OH, USA). Animals were mechanically ventilated to maintain an end-tidal CO_2 _to be within 4 to 5%, as monitored by a capnograph (Datex Normocap, Helsinki, Finland). All data were collected from animals with a maintained rectal temperature of 37 ± 0.5°C. At the end of each experiment, rats were killed with intravenous injection of an overdose of pentobarbital sodium (100 mg/kg).

### Induction of experimental endotoxemia

Experimental endotoxemia was induced by intravenous infusion (50 μl/min for 3 min) of *Escherichia coli*. lipopolysaccharide (LPS, 15 mg/kg, serotype 0111:B4; Sigma-Aldrich, St. Louis, MO, USA) to the anesthetized animals [26,27]. Infusion of the same amount of 0.9% saline served as vehicle and volume control. The temporal changes in mean SAP and HR were routinely followed for 8 h. Total survival time and survival rate, using 8 h postinfusion time interval as the cut-off time, were also recorded.

### Protein extraction and Western blot analysis

Extraction of total protein from the heart, lung or aorta was carried out as detailed previously [27]. In brief, tissue was lysed with ice-cold lysis buffer. Protease inhibitors (10 μg/ml aprotinin, 10 μg/ml leupeptin and 20 μg/ml phenylmethylsulfonyl fluoride) and phosphatase inhibitors (2 mM NaF, 1 mM sodium orthovanadate, 10 mM sodium pyrophosphate) were included in the lysis buffer to prevent protein degradation. Solubilized proteins were centrifugated at 20000 *g *at 4°C for 15 min, and proteins in the supernatant were quantified by the Bradford assay with a protein assay kit (Bio-Rad, Hercules, CA, USA).

Proteins (50 to 100 μg) were separated using 10% SDS-PAGE and transferred to PVDF membrane. The primary antiserum used for Western blot analysis included a rabbit polyclonal antiserum against neuronal NOS (nNOS), inducible NOS (iNOS), endothelial NOS (eNOS) (1:1000; BD Biosciences, San Jose, CA, USA) or α-tubulin (1:10000; Sigma-Aldrich). This was followed by incubation with horseradish peroxidase-conjugated goat anti-rabbit IgG (1:10000; Jackson Immunoresearch Laboratories, West Grove, PA, USA). Specific antibody-antigen complex was detected using an enhanced chemiluminescence Western Blot detection system (Perkin-Elmer Life Sciences, Boston, MA). The amount of detected protein was quantified by Photo-Print Plus software (ETS Vilber-Lourmat, France), and was expressed as the ratio to α-tubulin protein, which served as the internal control to demonstrate equal loading of proteins.

### Nitric oxide measurement

The tissue concentration of total nitrate and nitrite (NOx) was measured by chemical reduction-linked chemiluminescence using a purge system nitric oxide analyzer (Sievers NOA 280™, Boulder, CO, USA). Tissue homogenates of heart, lung or aorta were mixed in 0.4 N NaOH (0.3 ml) and were incubated at room temperature for 5 min. A 5% ZnSO_4 _(0.3 ml) was then added to the mixture and was incubated for another 5 min at room temperature. The mixture was centrifuged 3700 *g *for 20 min at 4°C, and the supernatant was injected into a purge vessel to react with the VCl_3_/HCl reagent, which converted nitrites and nitrates into NO. The amount of NOx in the test sample was determined by interpolation of the result into the standard curve. All assays were performed in triplicate and expressed as nmol/mg protein.

### Lipid peroxidation assay

Lipid peroxidation was quantified by determining malondialdehyde (MDA) level in the heart, lung or aorta via the thiobarbituric acid reacting substances (TBARS) [28]. The quantification of TBARS was determined by comparing the absorption at 532 nm to the standard curve of MDA equivalents generated by acid catalyzed hydrolysis of 1,1,3,3 tetramethoxypropane, and was expressed as nmol/mg protein.

### Oxidative burst activity

The extent of intracellular reactive oxygen species production was determined using a whole-blood assay in freshly drawn heparinized blood. The oxidative burst of polymorphonuclear leukocytes (PMN) or lymphocytes was determined using dihydroethidium (DHE) method. In brief, after oxidization step (30 min at 37°C) in which the non-fluorescent substrate, DHE, is taken up by the blood cells and converted into a fluorescent compound (ethidium) through respiratory burst metabolites, the citrated whole blood was lying, followed by white blood cell washing, and re-suspension in ice-cold PBS. The blood cell samples were gated and analyzed with a laser flow cytometer (FACScan/Lysis II, Becton Dickinson, Heidelberg, Germany) using blue/green excitation light (488 nm Argon Laser). Populations of monocytes and neutrophils were separately by electronic gating in the forward scatter (FSC)/side scatter (SSC) dot plot, and was measured and quantified in arbitrary units of 10,000 events.

### Experimental protocols

After a 30-min period of stable hemodynamics following the completion of general preparation, blood samples were collected from tail artery for baseline measurements. Animals received thereafter continuous intravenous infusion of propofol (AstraZeneca, Maccelesfield Cheshire, UK; 15 or 30 mg/kg/h) and were randomly assigned to receive LPS (endotoxemia group) or saline (control group) injection 60 min after propofol infusion. At 1, 4 or 6 h after the endotoxin, tissue (heart, lung and aorta) samples were harvested for analysis of NOS protein expression, NOx content, and lipid peroxidation. Blood samples were collected for determination of PMN or lymphocyte oxidative burst activity. This postinjection interval after the endotoxin was selected to represent the early or late phase endotoxemia, based on our previous reports [26,27] in which different phases of endotoxemia were characterized by changes in power density of the vasomotor components of SAP spectrum. To confirm the effect of propofol on LPS-evoked biochemical and hemodynamic responses, LPS was injection to animals that were maintained under pentobarbital sodium (Sigma-Aldrich, St Louis, MO; 15 mg/kg/h) anesthesia in a separate series of experiments. In our pilot study we found that pentobarbital sodium (15 mg/kg/h) provided satisfactory anesthetic maintenance similar to that by propofol (15 mg/kg/h).

### Statistical analysis

All values are expressed as mean ± SEM. One-way or two-way ANOVA with repeated measures was used, as appropriate, to assess group means. This was followed by the Scheffé multiple range test for post hoc assessment of individual means. A value of *P *< 0.05 was taken to indicate statistical significance.

## Results

### Effect of propofol on cardiovascular depression during endotoxemia

**Figure 1:**
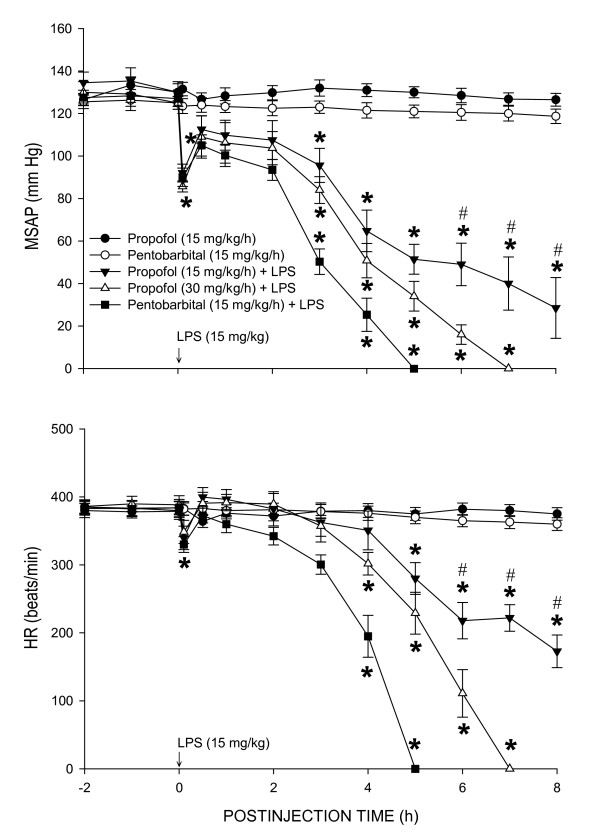
**Time-course changes in MSAP or HR after systemic injection of *Escherichia coli*. lipopolysaccharide (LPS, 15 mg/kg) or saline (n = 7) in rats that were maintained under propofol anesthesia (15 or 30 mg/kg/h; n = 7 or 8)**. Values are mean ± SEM, n = 7 or 8 animals per experimental groups. *P < 0.05 vs. propofol + saline group and ^#^P < 0.05 vs. low dose (15 m/kg/h) propofol group at corresponding time points in the Scheffé multiple-range test. The data of propofol (30 mg/kg/h) + saline group were not shown because they were similar to that of propofol (15 mg/kg/h) + saline group.

Baseline mean systemic arterial pressure (MSAP) or heart rate (HR) was comparable among the saline- or LPS-treated animals that received continuous infusion of propofol (15 or 30 mg/kg/h) (Fig. 1). In consistent to our previous findings [26,27], intravenous injection of LPS (15 mg/kg) caused transient but significant decreases in MSAP and HR, followed by partial recovery of the same hemodynamic parameters and the second phase of hypotension and bradycardia. The initial hypotension and bradycardia lasted less than 1 h and the delayed phase cardiovascular depression commenced approximately 3 h after the endotoxin. Compared to animals maintained under pentobarbital sodium (15 mg/kg/h) anesthesia, the LPS-induced second phase cardiovascular depression was dose-dependently blunted in those maintained under propofol (15 or 30 mg/kg/h) anesthesia (Fig. 1). Infusion of propofol or pentobarbital sodium alone, on the other hand, evoked no significant effect on baseline MSAP or HR during the 8-h observation period.

### Effect of propofol on tissue lipid peroxidation during endotoxemia

**Figure 2:**
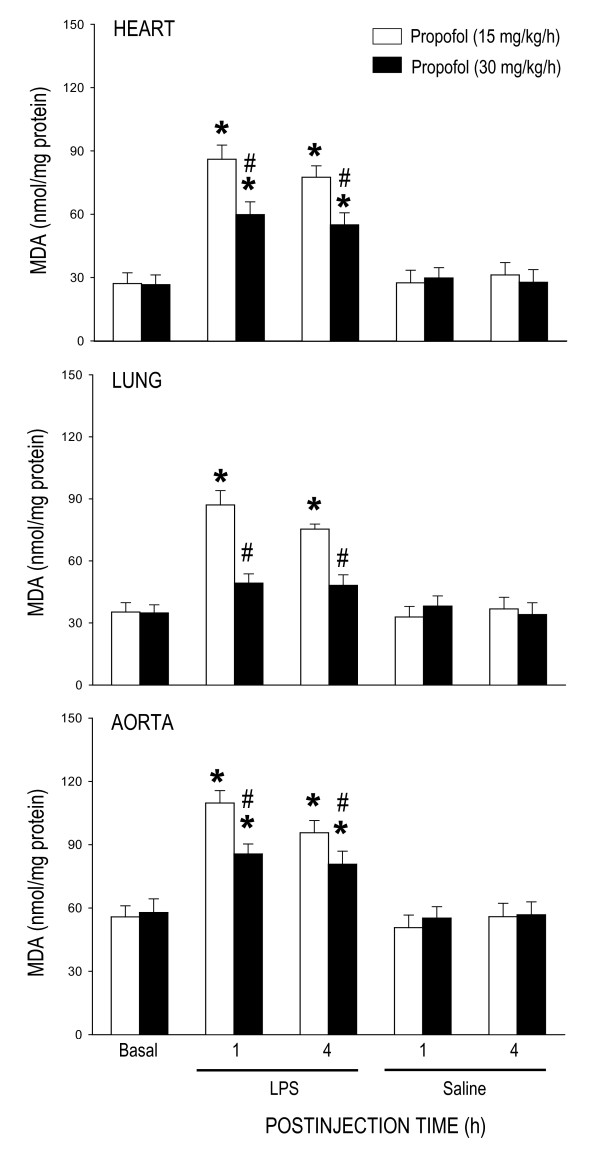
**Changes in tissue level of malondialdehyde (MDA) in the heart, lung or aorta at 1 or 4 h after systemic injection of LPS (15 mg/kg) or saline in rats that were maintained under propofol anesthesia (15 or 30 mg/kg/h)**. Values are mean ± SEM of quadruplicate analyses of samples from 4 animals per each experimental group. *P < 0.05 vs. saline group and ^#^P < 0.05 vs. low dose (15 m/kg/h) propofol group at corresponding time points in the Scheffé multiple-range test. Basal indicates MDA level in the corresponding tissue prior to LPS injection. The same data prior to saline injection was not shown because they were similar to that of LPS groups.

The change in tissue MDA production was used in the present study to reflect lipid peroxidation. While propofol anesthesia (15 or 30 mg/kg/h) alone did not affect basal level of MDA, the LPS-induced increase in MDA production in the heart, lung or aorta was significantly ameliorated in animals that received propofol infusion at 30 mg/kg/h (Fig. 2). This protective effect by propofol was observed during initial (i.e., 1 h postinjection) and delayed (i.e., 4 h postinjection) phases of endotoxemia. Moreover, LPS-induced lipid peroxidation was almost completely prevented in the lung of animal maintained under propofol infusion at 30 mg/kg/h (Fig. 2). At 6 h after LPS injection, protection by high dose (30 mg/kg/h) propofol on the LPS-induced increases in tissue MDA production in the heart (69.8 ± 7.3 nmol/mg protein, n = 6), lung (63.8 ± 5.1 nmol/mg protein, n = 6) or aorta (89.0 ± 6.4 nmol/mg protein, n = 6) was diminished. Systemic injection of LPS resulted in a grater increase in MDA production detected at 4 h postinjection in the heart (144.9 ± 10.7 nmol/mg protein, n = 4), lung (138.6 ± 10.6 nmol/mg protein, n = 4) or aorta (146.4 ± 11.6 nmol/mg protein, n = 4) of animals maintained under pentobarbital sodium anesthesia. This anesthetic agent alone did not affect basal level of MDA in the same tissues (heart: 25.9 ± 4.8 nmol/mg protein, n = 4; Lung: 37.9 ± 6.4 nmol/mg protein, n = 4; aorta: 60.4 ± 5.8 nmol/mg protein, n = 4).

### Effect of propofol on oxidative respiratory burst of blood cells during endotoxemia

**Figure 3:**
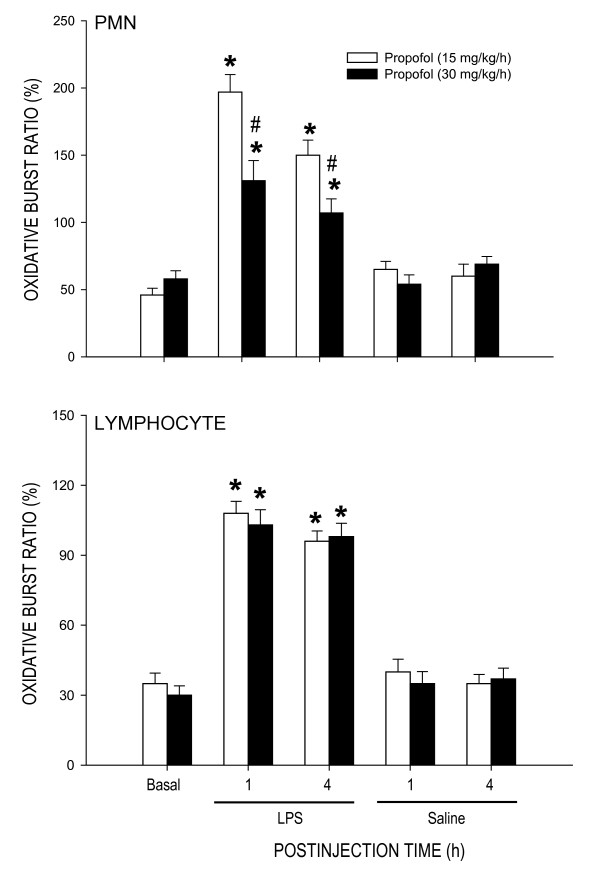
**Change in oxidative burst ratio of polymorphonuclear leukocytes (PMN) or lymphocytes at 1 or 4 h after systemic injection of LPS (15 mg/kg) or saline in rats that were maintained under propofol anesthesia (15 or 30 mg/kg/h)**. Values are mean ± SEM of quadruplicate analyses of samples from 4 animals per each experimental group. *P < 0.05 vs. saline group and ^#^P < 0.05 vs. low dose (15 m/kg/h) propofol group at corresponding time points in the Scheffé multiple-range test. Basal indicates oxidative burst ratio in the corresponding tissue prior to LPS injection. The same data prior to saline injection was not shown because they were similar to that of LPS groups.

LPS treatment (15 mg/kg) also resulted in oxidative respiratory burst of PMN and lymphocytes from the blood of animals under propofol anesthesia (Fig. 3). Compared to propofol anesthesia at 15 mg/kg/h, LPS-induced increase in oxidative burst of PMN, but not lymphocytes, was significantly reduced at 1 or 4 h after the endotoxin in animals that received propofol anesthesia at 30 mg/kg/h. Propofol alone, at either dose, did not affect the ROS production in PMN or lymphocytes after saline injection.

### Effect of propofol on tissue nitric oxide concentration during endotoxemia

**Figure 4:**
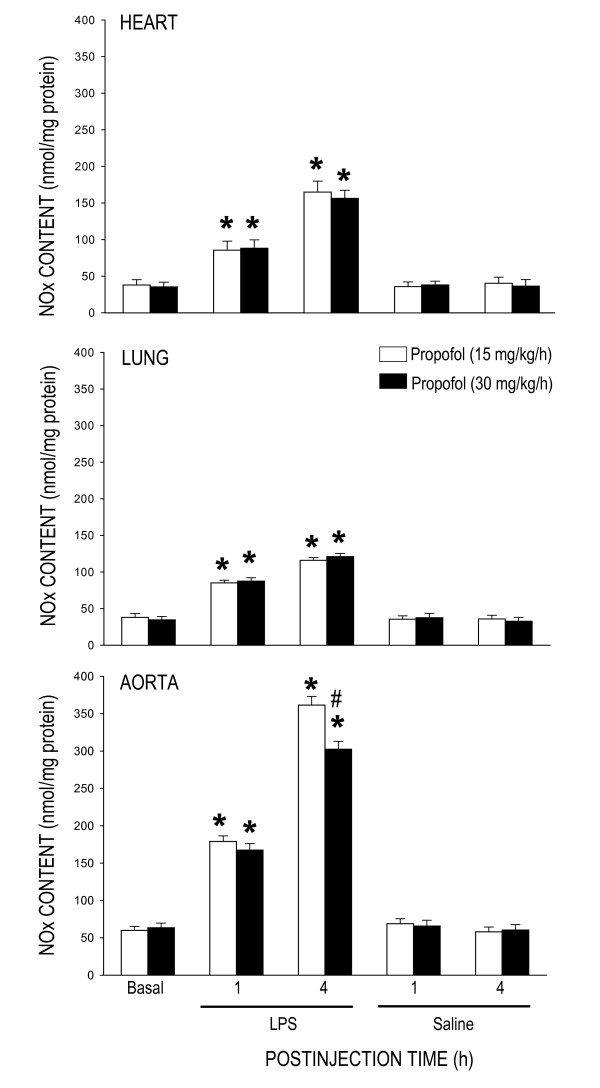
**Changes in tissue level of nitrite and nitrate (NOx) in the heart, lung or aorta at 1 or 4 h after systemic injection of LPS (15 mg/kg) or saline in rats that were maintained under propofol anesthesia (15 or 30 mg/kg/h)**. Values are mean ± SEM of quadruplicate analyses of samples from 4 animals per each experimental group. *P < 0.05 vs. saline group and ^#^P < 0.05 vs. low dose (15 m/kg/h) propofol group at corresponding time points in the Scheffé multiple-range test. Basal indicates NOx content in the corresponding tissue prior to LPS injection. The same data prior to saline injection was not shown because they were similar to that of LPS groups.

Compared with saline control, LPS treatment (15 mg/kg) significantly increased NOx content in the heart, lung or aorta, measured at 1 or 4 h after the endotoxin, in animals under propofol anesthesia (15 or 30 mg/kg/h) (Fig. 4). Similar results were obtained in the heart (130 ± 15 nmol/mg protein, n = 4), lung (119 ± 11 nmol/mg protein, n = 4) or aorta (366 ± 23 nmol/mg protein, n = 4) 4 h after systemic injection of LPS to animals maintained under pentobarbital sodium anesthesia. The LPS-induced NOx surges in the heart and lung during the initial and delayed phases of endotoxemia were comparable in animals that received propofol anesthesia at 15 or 30 mg/kg/h. At 6 h after high dose (30 mg/kg, i.v.) propofol infusion, NOx levels in the heart (152 ± 13 nmol/mg protein, n = 6) or lung (118 ± 11 nmol/mg protein, n = 6) was comparable to that detected in 1 or 4 h after LPS injection. The increase in NOx content in the aorta during the delayed, but not initial, phase of endotoxemia was discernibly blunted in animals under propofol anesthesia at 30 mg/kg/h (Fig. 4). This protection against NOx surge by high dose propofol (270 ± 19 nmol/mg protein, n = 6) lasted for at least 6 h after LPS injection. Propofol (Fig. 4) or pentobarbital sodium infusion alone had no effect on tissue level of NOx in the heart (39.8 ± 5.4 nmol/mg protein, n = 4), lung (40.2 ± 4.3 nmol/mg protein, n = 4) or aorta (65.0 ± 6.5 nmol/mg protein, n = 4) after saline injection.

### Effect of propofol on protein expression of nitric oxide synthase during endotoxemia

**Figure 5:**
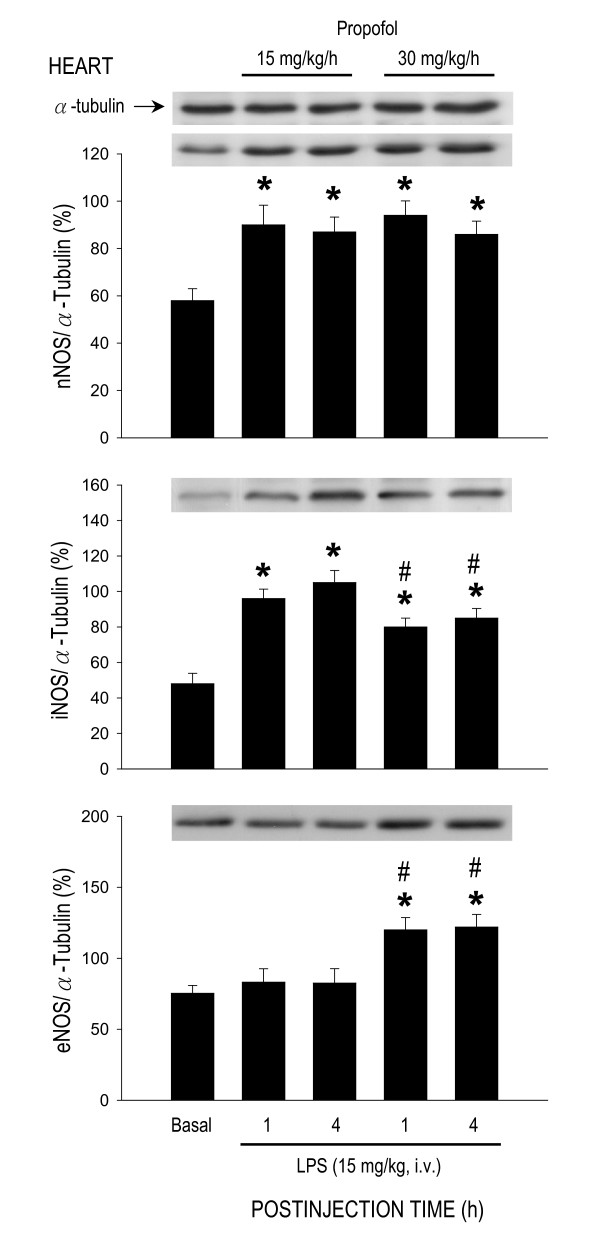
**Representative Western blots of neuronal nitric oxide synthase (nNOS), inducible NOS (iNOS) or endothelial NOS (eNOS) (insets) or densitometric analysis in amount of protein relative to α-tubulin from the heart at 1 or 4 h after systemic injection of LPS (15 mg/kg) in rats that were maintained under propofol anesthesia (15 or 30 mg/kg/h)**. Values are mean ± SEM of quadruplicate analyses of samples from 4 animals per each experimental group. *P < 0.05 vs. basal expression and ^#^P < 0.05 vs. low dose (15 m/kg/h) propofol group at corresponding time points in the Scheffé multiple-range test. Basal indicates expression of nNOS, iNOS or eNOS in the heart of the control groups at 30 min after propofol infusion.

**Figure 6:**
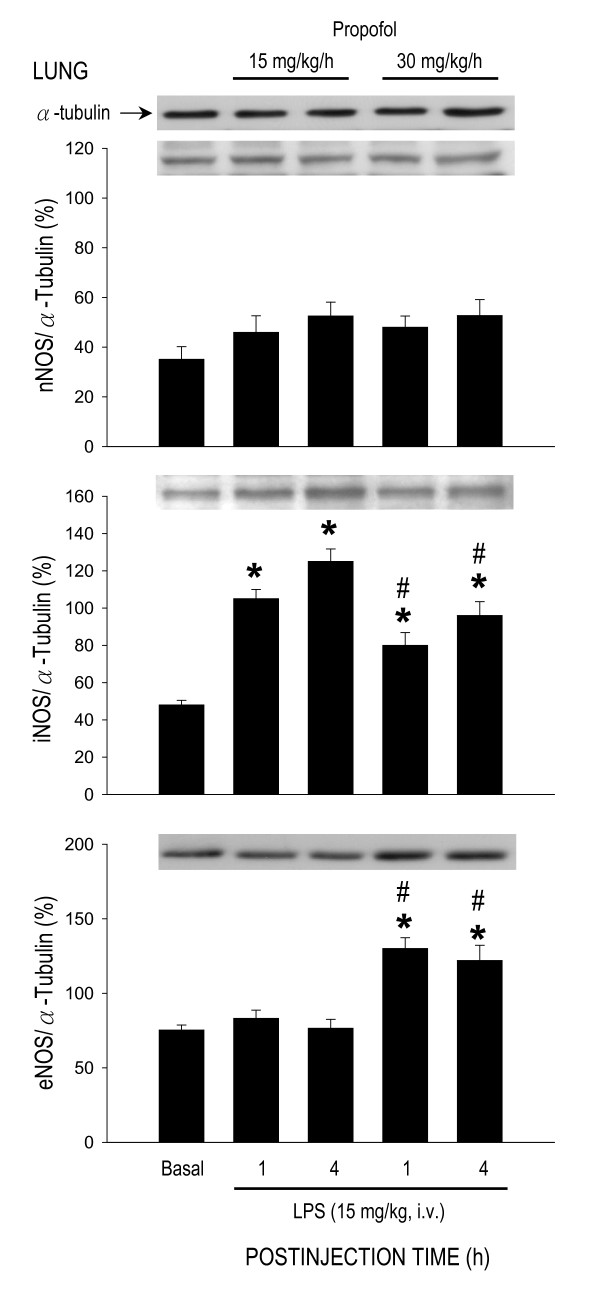
**Representative Western blots of nNOS, iNOS or eNOS (insets) or densitometric analysis in amount of protein relative to α-tubulin from the lung at 1 or 4 h after systemic injection of LPS (15 mg/kg) in rats that were maintained under propofol anesthesia (15 or 30 mg/kg/h)**. Values are mean ± SEM of quadruplicate analyses of samples from 4 animals per each experimental group. *P < 0.05 vs. basal expression and ^#^P < 0.05 vs. low dose (15 m/kg/h) propofol group at corresponding time points in the Scheffé multiple-range test. Basal indicates expression of nNOS, iNOS or eNOS in the lung of the control groups at 30 min after propofol infusion.

**Figure 7:**
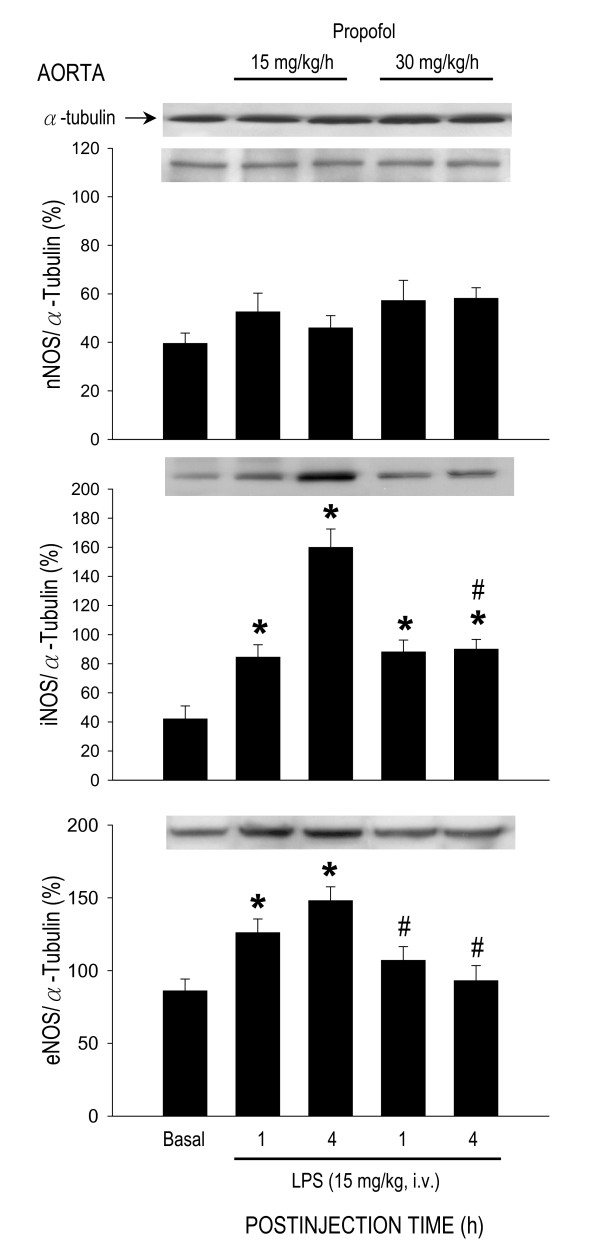
**Representative Western blots of nNOS, iNOS or eNOS (insets) or densitometric analysis in amount of protein relative to α-tubulin from the aorta at 1 or 4 h after systemic injection of LPS (15 mg/kg) in rats that were maintained under propofol anesthesia (15 or 30 mg/kg/h)**. Values are mean ± SEM of quadruplicate analyses of samples from 4 animals per each experimental group. *P < 0.05 vs. basal expression and ^#^P < 0.05 vs. low dose (15 m/kg/h) propofol group at corresponding time points in the Scheffé multiple-range test. Basal indicates expression of nNOS, iNOS or eNOS in the aorta of the control groups at 30 min after propofol infusion.

LPS treatment (15 mg/kg) also induced differential effects on protein expression of NOS isoforms in the heart, lung or aorta. In the heart (Fig. 5), but not lung (Fig. 6) or aorta (Fig. 7), expression of nNOS protein was significantly increased by LPS treatment in animals that were under propofol anesthesia (15 or 30 mg/kg/h). The endotoxin, on the other hand, induced an upregulation of iNOS expression in all three organs during early and late phases of endotoxemia (Figs. 5, 6, 7). The LPS-induced iNOS upregulation in both phases of endotoxemia was significantly attenuated in the heart (Fig. 5) or lung (Fig. 6) of animals that received propofol anesthesia at 30 mg/kg/h. The same anesthetic infusion only inhibited iNOS upregulation in the aorta during late stage endotoxemia (Fig. 7). LPS treatment also induced eNOS upregulation in the heart (Fig. 5) or lung (Fig. 6) during both phases of endotoxemia in animals that were maintained under propofol anesthesia at 30 mg/kg/h. The endotoxin, on the other hand, induced eNOS expression in the aorta of animals that received propofol infusion at 15 mg/kg/h (Fig. 7). Propofol alone, at either dose, did not affect basal expression of nNOS, iNOS or eNOS in the heart, lung or aorta of animals that received saline injection (data not shown).

### Effect of propofol on survival time and mortality after LPS treatment

**Figure 8:**
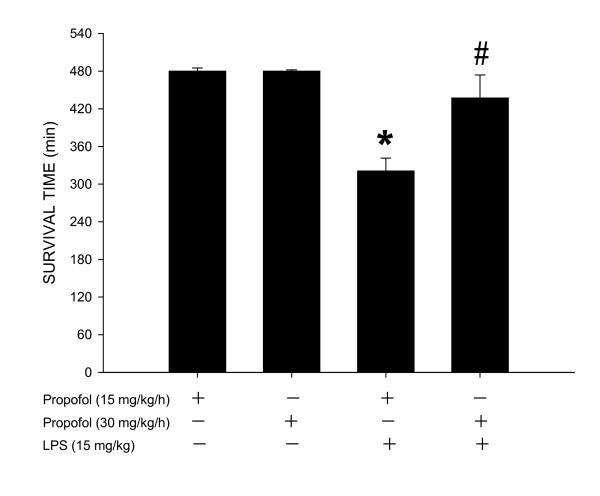
**Changes in survival time during 8-h observation period after systemic injection of LPS (15 mg/kg) or saline (n = 8) in rats that were maintained under propofol anesthesia (15 or 30 mg/kg/h; n = 9 or 8)**. Values are mean ± SEM, n = 8 or 9 animals per experimental groups. *P < 0.05 vs. propofol group and ^#^P < 0.05 vs. low dose propofol (15 m/kg/h) group in one way ANOVA.

Compared to low dose (15 mg/kg/h) propofol infusion, survival time after LPS treatment (15 mg/kg) was significantly longer in animals that were maintained under high dose (30 mg/kg/h) propofol anesthesia (Fig. 8). Mortality rate 8 h after endotoxin injection was 100% or 60%, respectively, for animals under propofol anesthesia at 15 or 30 mg/kg/h. All animals that were under propofol anesthesia (15 or 30 mg/kg/h) survived the entire 8-h observation period after saline injection.

## Discussion

Compelling evidence indicates that cardiovascular depression plays a pivotal role in pathological sequelae leading to multiple organ failure and fatality during endotoxemia. Both oxidative injury and overproduction of NO in cardiovascular and pulmonary systems contribute to fatal cardiovascular depression during endotoxemia [4-7,11,12,16]. We provided novel evidence in the present study to suggest that oxidative injury and NO may play a differential role in LPS-induced cardiovascular depression. ROS is associated with both early and late stage; whereas NO is engaged primarily in late stage cardiovascular depression. We further revealed that propofol infusion may protect against fatal cardiovascular depression during endotoxemia by attenuating the late stage NO surge in the aorta, possibly via inhibition of iNOS upregulation by the endotoxin.

Diverse molecular mechanisms of cellular damage by the endotoxin contribute to cardiovascular depression during sepsis, of which overt production of NO and oxidative injury to cardiovascular and pulmanory organs are of praticular importance [6-8]. Although a vast amount of evidence supports the pivatol roles of NO and ROS in pathophysiology of endotoxemia, relative contribution of these molecules in various organs during different stages of cardiovascular depression has not been fully defined. As such, one major contribution of the present study is to demonstrate a differential association of oxidative tissue injury and NO surge in LPS-induced cardiovascular depression. In consistent to our previous findings [26,27], LPS (15 mg/kg, i.v.) induces a biphasic cardiovascular depression during endotoxemia. The early hypotension appeared immediately and lasted for approximately 1 h after LPS injection and the late hypotension commenced 3 h after the endotoxin injection. In the heart, lung and aorta, an abrupt increase in lipid peroxidation, our experimental index of oxidative tissue injury, was detected in early stage and sustained during late stage cardiovascular depression. LPS injection, on the other hand, induced a gradual increase in tissue nitrite and nitrate level in the same organs that peaked during late stage endotoxemia. Intriguingly, whereas the heart (maximal increase in MDA production: 315 ± 18%, n = 4) and lung (maximal increase in MDA production: 247 ± 23%, n = 4) are more vulnerable than the aorta (maximal increase in MDA production: 189 ± 17%, n = 4) to oxidative injury by the endotoxin (cf. Fig. 2); NO production induced by LPS is profoundly augmented in the aorta (maximal increase in NOx level: 587 ± 45%, n = 4) than the heart (maximal increase in NOx level: 329 ± 31%, n = 4) or the lung (maximal increase in NOx level: 436 ± 39%, n = 4) (cf. Fig. 4). Together these results suggest that ROS and NO may differentially mediate the deleterious effects on different cardiovascular and pulmonary organs during different stages of endotoxemia. In support of this suggestion, oxidative stress of an increase tissue level of O_2_^•- ^is greater in the lung than aorta during early and late stage endotoxemia induced by a cecal ligation and puncture [29]. A gradual increase in plasma [30] or myocardial [31] concentration of nitrates/nitrites was evident during late stage endotoxemia. Pathologically, ROS production in the lung impairs pulmonary vascular function early in the course of endotoxemia [32]. A sustained oxidative damage to the aorta is involved in hypotension [23,29] and endothelial dysfunction [33,34] during late stage endotoxemia. In addition, early and delayed production of O_2_^•- ^via activation of the NADPH oxidase mediates myocardial depression in LPS-treated heart [35]. Vascular hyporeactivity to vasoactive compound [23], delayed hypotension [29] and inflammation of lung tissue [36,37], on the other hand, are attributed to overproduction of NO during late stage of endotoxemia.

Patients with endotoxemia often require drugs for sedation and analgesia in the intensive care units. Several anesthetics, such as ketamine [38], dexmedetomidine [39] and propofol [39,40], have been used in patients with septic shock for these purposes. Whether propofol exerts protection against cardiovascular depression during endotoxemia, however, is still debatable. In animal model of sepsis, propofol has been shown to exert protection against [41], no significant effect [24,36] or even aggravation [36] on cardiovascular depression during endotoxemia. In the present study we demonstrate that propofol exerted a dose-dependent differential protection against cardiovascular depression during different stage of endotoxemia. During early stage endotoxemia, there was no apparent difference in cardiovascular response to the endotoxin in rats subjected to low (15 mg/kg) or high (30 mg/kg) dose propofol infusion. High dose propofol, on the other hand, ameliorated cardiovascular depression during late stage endotoxemia. It was reported that the biphasic cardiovascular responses to endotoxemia were comparable in endotoxemic rats that received propofol infusion at 5, 10, or 15 mg/kg [22,24,36]. Although we did not estimate plasma concentration of propofol, the reported mean blood propofol concentration after infusion of propofol at 10, 15 or 60 mg/kg into rats was 4.2 ± 0.4 μg/ml [24], 8.2 ± 1.9 μg/ml [36] or 12.4 ± 0.8 μg/ml [42], respectively. These plasma concentrations exceed the propofol plasma concentration required to produce anesthesia [43,44]. In this regard, the LPS-evoked cardiovascular depression was more severe in animals supplemented with pentobarbital sodium, which provided satisfactory anesthesia similar to that by propofol. Together these results suggest that the anesthetic effect of propofol do not contribute primarily to cardiovascular protection during endotoxemia and that a much higher plasma concentration of propofol may need to exert cardiovascular protection during endotoxemia. We realize that discrepancy on cardiovascular protective effect of propofol on endotoxemia may also depend on animal species (rodent, porcine, or rabbit) and animal models (cecal ligation and puncture, LPS injection or) of endotoxemia, as well as routes (subcutaneous, intraperitoneal, intravenous) and regimens (pre- or post-LPS treatment) of drug application.

Our results indicate that inhibition of late stage NO surge in the aorta by propofol may underlie protection against fatal cardiovascular depression during endotoxemia by the anesthetic. Overproduction of NO via activation of iNOS contributes to fatal cardiovascular depression and mortality during late stage endotoxemia [45,46]. Propofol at dose (30 mg/kg/h) that significantly attenuated the late stage NO surge in the aorta at 4 and 6 h after LPS injection also ameliorated cardiovascular depression during the same stage of endotoxemia. A lack of effect to reverse LPS-induced NO production in the heart, lung or aorta during early stage endotoxemia, alongside an insignificant effect of propofol on hemodynamic suppression during the same stage of endotoxemia provide further support to the suggestion. Moreover, low dose (15 mg/kg/h) propofol that did not affect the LPS-induced early or late NO surge also did not protect against cardiovascular depression during early and late stage of endotoxemia. Although plasma level of NOx was not measured in the present study, it was reported that LPS causes at least 3 fold increase in plasma NOx levels during the first 6 h after the endotoxin, and propofol infusion significantly decreases plasma concentration of NOx [24,47]. Our results also imply that the anti-oxidant effect of propofol may not play an active role in its cardiovascular protective effect during endotoxemia. We found that although suppressing significantly the LPS-induced lipid peroxidation in the heart, lung and aorta, as well as ROS production in the PMN of the blood during early stage endotoxemia, high dose propofol did not affect the cardiovascular depression during the same stages of endotoxemia. Moreover, suppression of ROS production in the same organs by high dose propofol actually blunted at 6 h after LPS injection during which protection of cardiovascular depression occurred. Together these results suggest that propofol may exert protection against late stage fatal cardiovascular depression primarily by suppression of NO surge, although its anti-oxidant effect during late stage endotoxemia could not be excluded.

Of the three NOS isoforms, our results demonstrated that inhibition of LPS-induced iNOS upregulation by high dose propofol may underlie the reduction in NO surge in the aorta during late stage endotoxemia. It is well documented that iNOS-derived NO in the heart [48], lung [37] and aorta [45] contributes respectively to myocardial dysfunction, acute lung injury and systemic hypotension associated with endotoxemia. Inhibition of iNOS activity or iNOS induction, on the other hand, attenuates the delayed circulatory failure during endotoxemia [46]. Propofol has been reported to inhibit the induced iNOS expression by endotoxin [49]. In the present study we found that only in late stage endotoxemia there was a close association between inhibitions of LPS-induced iNOS upregulation, reduction in plasma NOx levels in the aorta with amelioration of cardiovascular depression by high dose propofol. It is interesting to note that inhibition by propofol in the LPS-induced iNOS upregulation in the heart or lung was not accompanied by attenuation in NO surge during early and late endotoxemia. These results were interpreted to suggest that iNOS might not contribute significantly to the increased NO production in the heart and lung by LPS. In this regard, in murine sepsis-induced acute lung injury, pulmonary oxidant stress is completely iNOS dependent and is associated with tyrosine nitration [50]. The iNOS also mediates the nitrosative/oxidative damage and cardiac mitochondrial dysfunction that occurs during endotoxemia [51]. Alternatively, our finding of the insignificant change in LPS-induced NO surge in the heart or lung during endotoxemia might be the consequences to the upregulations of eNOS expression by high dose propofol.

In contrast to iNOS, a potential role for eNOS in cardiovascular depression during endotoxemia is controversial. The LPS-induced increase in plasma level of nitrite and nitrate was reported to be identical [52] or reduced [47] in eNOS^-/- ^knockout mice. Moreover, mice overexpressing eNOS transgene generate similar levels of plasma nitrite and nitrate to control animals in response to LPS [53]. Conflict results also exist in LPS-induced cardiovascular depression and fatality, varying from no significant difference [52] to resistance [47,53,54] to endotoxemic shock in eNOS^-/- ^knockout mice. In the present study we found that eNOS expressions were upregulated in the heart and lung during early and late stage endotoxemia in rats subjected to high dose propofol infusion. Since iNOS may inhibit eNOS expression during endotoxemia [55], we speculate that eNOS upregulations in these organs might be a compensatory change to inhibition of the LPS-induced iNOS expression during endotoxemia by high dose propofol. Propofol inhibits the iNOS induction via suppression of NF-κB nuclear translocation [56]. Induction of eNOS transcript is regulated by PI3K/Akt-dependent pathway in endothelial cells [57]. Whether these mechanisms account for the differential effects of propofol on iNOS and eNOS expression during endotoxemia, however, await further investigation. We also fund in the present study that high dose propofol resulted in eNOS upregulation in the heart and lung, but not in the aorta, during early and late stage endotoxemia. The underlying mechanism of these discrepancies is not immediate clear. Propofol has been demonstrated to exert various effects on eNOS expression under different pathological conditions. The eNOS expression is increased by propofol in hydrogen peroxide-stimulated [58] but inhibited in LPS-stimulated human umbilical vein endothelial cells [59]. Propofol, on the other hand, had no effect on testicular endothelial cells during ischemia-reperfusion injury [49].

The role of nNOS in cardiovascular failure and mortality of sepsis also remains highly elusive. In nNOS^-/- ^knockout mice, mortality is increased in sepsis, possibly by increasing proinflammatory cytokine response and impairing bacterial clearance [60]. In contrast, deletion of nNOS prevents impaired vasodilation [61] and restores arteriolar vasoconstriction in sepsis [62]. Although we found in the present study that nNOS expression in the heart was significantly increased during early and late stage endotoxemia, this induced nNOS upregulation was, nonetheless, not affected by propofol infusion. Significance of nNOS in cardiovascular depression during endotoxemia, therefore, remains to be elucidated.

In addition to oxidative injury of cardiovascular and pulmonary organs, we found significant increases in respiratory burst activity in the peripheral blood PMN and lymphocytes during early and late stage endotoxemia. The PMN and lymphocytes play central roles in LPS-induced inflammatory response [7,44]. Reduced neutrophil and lymphocyte function, as determined by the increase in respiratory burst activity, may therefore lead to persistence of infection, resulting in the maintenance of septic shock, multiple organ dysfunction and death [44]. Our results of inhibition by high dose propofol in LPS-induced early and late phases of oxidative burst of PMN are in consistent to the previous reports [7,44], and suggest the beneficial effect of propofol to endotoxemia. This inhibition, nonetheless, may not contribute significantly to cardiovascular protection by high dose propofol. We found that despite of protection on LPS-induced augmentation in PMN burst reaction during early stage endotoxemia, high dose propofol did not protect against cardiovascular depression during the same stage of endotoxemia. A differential protective effect of high dose propofol on oxidative burst in PMN versus lymphocytes is intriguing. Since PMN is more prone than lymphocytes to oxidative damage during endotoxemia, these blood cells might also be more sensitive to agents that protect against the oxidative burst.

We notice that the dose (15 or 30 mg/k/h) of propofol used in the present study was higher than the reported dose (5 or 10 mg/kg/h) on protection against LPS-induced inflammatory response [19] or metabolic acidosis [44], but much lower than that (120 mg/kg/h) to reduce susceptibility of the red blood cells to oxidative damage [63]. High dose propofol has been reported to evoke a direct cardiovascular suppression via inhibition of protein kinase C-mediated contraction of vascular smooth muscles [23]. It is empirical to include animals that received LPS injection only as the control. Since the experimental endotoxemia model used in the present study was induced under anesthetic condition, the LPS alone group (without propofol anesthesia) does not follow the ethical guideline for care and use of laboratory animals. Nonetheless, we have included saline-treated group to demonstrate that propofol alone did not affect NOx, NOS expression, ROS production or basal hemodynamics (cf. Figs. 1, 2, 3, 4). We therefore reasoned that the protective effects by propofol were observed only under endotoxemic condition. We also realize that the propofol preparation used in this study contains lipid components that may exert biological activity. This possibility, however, is deem unlikely since propofol infusion alone did not affect NOx, NOS expression, PMN burst reaction or hemodynamic parameters in saline-treated animals. In addition, lipid component (intralipid) of propofol was reported to evoke minimal effects on NO and ROS production [23].

## Conclusion

In conclusion, we have demonstrated that oxidative injury and NO may play a differential role in LPS-induced cardiovascular depression during endotoxemia. ROS is associated with both early and late stage; whereas NO is engaged primarily in late stage cardiovascular depression. We further revealed that propofol infusion may protect against fatal cardiovascular depression during endotoxemia by attenuating the late stage NO surge in the aorta, possibly via inhibition of iNOS upregulation by the endotoxin. These beneficial effects of propofol may contribute to the higher survival rate of rats with endotoxemia.

## Competing interests

The authors declare that they have no competing interests.

## Authors' contributions

YCL conceived the study, carried out animal experiments, performed biochemical analysis, collected experimental data, performed the statistical analysis and interpretation of data, and drafted the manuscript. AYWC participated in experimental design and was involved in revising the manuscript for important intellectual content. YCT participated in experimental design and coordination, and was involved in revising the manuscript for important intellectual content. JYHC participated in experimental design, interpretation of the data, involved in revising the manuscript for important intellectual content and have given final approval of the version to be published. All authors read and approved the final manuscript.
